# Two maize cultivars of contrasting leaf size show different leaf elongation rates with identical patterns of extension dynamics and coordination

**DOI:** 10.1093/aobpla/plaa072

**Published:** 2021-01-04

**Authors:** Tiphaine Vidal, Hafssa Aissaoui, Sabrina Rehali, Bruno Andrieu

**Affiliations:** UMR ECOSYS, INRAE, AgroParisTech, Université Paris-Saclay, Thiverval-Grignon, France

**Keywords:** Blade, coordination, emergence, extension rate, initiation, leaf, phyllochron, phytomer, sheath, Zea mays

## Abstract

Simulating leaf development from initiation to maturity opens new possibilities to model plant–environment interactions and the plasticity of plant architecture. This study analyses the dynamics of leaf production and extension along a maize (*Zea mays*) shoot to assess important modelling choices. Maize plants from two cultivars originating from the same inbred line, yet differing in the length of mature leaves were used in this study. We characterized the dynamics of the blade and sheath lengths of all phytomers by dissecting plants every 2–3 days. We analysed how differences in leaf size were built up and we examined the coordination between the emergence of organs and phases of their extension. Leaf extension rates were higher in the cultivar with longer leaves than in the cultivar with shorter leaves; no differences were found in other aspects. We found that (i) first post-embryonic leaves were initiated at a markedly higher rate than upper leaves; (ii) below ear position, sheaths were initiated at a time intermediate between tip emergence and appearance, while above the ear position, sheaths were initiated at a high rate, such that the time interval between the blade and sheath initiations decreased for these leaves; and (iii) ear position also marked a change in the correlation in size between successive phytomers with little correlation of size between upper and lower leaves. Our results identified leaf extension rate as the reason for the difference in size between the two cultivars. The two cultivars shared the same pattern for the timing of initiation events, which was more complex than previously thought. The differences described here may explain some inaccuracies reported in functional–structural plant models. We speculate that genotypic variation in behaviour for leaf and sheath initiation exists, which has been little documented in former studies.

## Teaser

We compared leaf development and extension in two maize cultivars with same genetic background but differing in their size. We showed coordinations linking the initiation, differentiation and extension of leaves with their emergence. Some coordinations markedly differ before and after tassel initiation, resulting in successive leaves increasing in length up to ear position and then decreasing in length. Both cultivars showed remarkably similar coordinations, yet differed by an higher leaf extension rates in the taller cultivar. The work contributes to clarify the features that organize the timing of events and the pattern of successive leaf sizes along a maize shoot.

## Introduction

Simulating the development of leaf surfaces is an essential component of crop and plant models. Many crop models simulate the development of leaf area index by a global equation (e.g. CERES, STICS, SIRIUS: see Boote *et al.* 2013 for a review). In contrast, functional–structural plants models (FSPMs), and a few crop models (Porter 1993; Lizaso *et al.* 2011) capture the successive development and senescence of individual leaves along a plant shoot. Simulating leaf development implies additional complexity compared with simulating global leaf surface; however, it allows for a more precise expression of a large range of plant–environment interactions, such as which organs are exposed to environmental stressors or to protective treatments, and how plants respond to stressors or stimuli. The size of mature leaves is largely dependent on growth conditions early in their extension when these leaves represented a negligible part of exposed surfaces. Describing the full sequences of leaf extension from its initiation to its full unfolding, as done in some FSPMs (Gauthier *et al.* 2020), is thus a pre-requisite to precisely relate the plant’s exposure to stressors to its response. A plant’s response to stress is largely delayed in time and involves leaves that were small but growing at the time of stress.

Monocots are a large group of species in which a long early phase of leaf growth occurs when the leaf is hidden in a tube made by sheaths of older leaves. From the numerous studies that characterized the extension of monocot leaves, especially cereals and forage grasses, it appears that the same growth scheme is widely shared among species (Freeling 1992; Fournier *et al.* 2005). After the initiation of the primordium by the stem apex, the leaf successively establishes several regions organized along an acropetal gradient: cell division, cell extension, and mature tissues. The growth zone (extension and division) extends inside the sheath tube and its maximal length is approximately two-thirds of it (Kemp 1980). Tissues mature in the remaining part of the tube and are exposed to full light only after they have completed their development. In terms of the dynamics of extension, the establishment of the division zones and the elongation zone correspond to exponential kinetics of the increase in leaf length vs. time, while the production of mature tissues corresponds to a quasi-linear extension, as the growth zone is approximately stabilized in length. Thus, leaf extension can be descriptively modelled as a succession of one or two exponential phases, and a linear phase that starts approximately when the leaf tip is exposed from the sheath tube. Leaf extension stops when the leaf sheath is exposed (Durand *et al.* 1999; Fournier *et al.* 2005; Zhu *et al.* 2014).

There is a consensus on an overall process that coordinates the phases of extensions with the time of organ exposure. This coordination has been the basis for various models of grass leaf extension. Existing models, however, differ in important aspects. In most models, the timing of the emergence of leaf tips and ligules is externally imposed, based on the empirical evidence that these events occur at regular time intervals. In such models, the parameters of leaf extension are adjusted so that the emergence follows a predetermined timetable. In a few models (Zhu *et al.* 2014; Gauthier *et al.* 2020; Vidal *et al.* 2020), the emergence of leaf tips and ligules is calculated according to the organ extension compared with the dimension of older organs, so that the phyllochron and phases of leaf extension are dynamically regulated and depend on leaf initiations and growth rates. The latter approach brings the promise of understanding how patterns in size and time emerge from extension and coordination. Indeed, the approach allowed for predicting some observed patterns; however, the behaviour of these models depends on the values of their parameters and on the stability of coordination along the shoot, for which information is scarce. Most research on this topic has been conducted in maize; nonetheless, significant information is missing.

For instance information on how the sheath/blade ratio is regulated remains incomplete. The sheath/blade boundary can first be observed as a line of cells that differentiates early in leaf development (Freeling 1992) then passively moves through the leaf growing-zone (Kemp 1980), thus delimiting which regions are dedicated to the division and extension of blade and sheath cells across time. Ultimately, the differentiation of the sheath/blade boundary regulates leaf length and the sheath/blade ratio. Consequently, the rules for the timing of sheath initiation play an important role in models that mechanistically address leaf extension and the blade/sheath ratio. Several studies have addressed the genetic control of this proximal–distal differentiation (reviewed in Bolduc *et al.* 2012, Richardson *et al.* 2018, Satterlee *et al.* 2019) and have associated it with the spatial/temporal regulation of the *OsBOPS* gene (Toriba *et al.* 2019). However, it is unknown how the activity of *OsBOPS* is regulated. In various grass species, several studies have reported quasi-synchronicity between leaf appearance and start of sheath–blade differentiation (Silvy 1982*a*, *b*; Skinner *et al.* 1994; Andrieu *et al.* 2006) leading to the interpretation that tip exposure to light and/or atmosphere may be what triggers the differentiation of the sheath-blade boundary. There are, however, at least two distinct candidate events related to the triggering of the differentiation of the sheath-blade boundary: (i) the leaf tip emergence above the sheath tube, which corresponds to the exposure of leaf tip to the atmosphere and (ii) the leaf tip appearance out of the whorl, which corresponds to the exposure of the leaf tip to full light and occurs after leaf emergence. These two events are nearly synchronous in grasses with a short whorl, such as wheat or grassland species, for which most of the reports for the synchronisms with the sheath differentiation have been established. In contrast, leaf appearance is significantly delayed after leaf emergence in species, such as maize or sorghum, in which the sheath tube is continued by a long whorl made of rolled blades. Although there is an abundance of data on the rate of leaf appearance in these species, there is a lack of data on leaf emergence. Leaf appearance is easy to monitor, but is difficult to simulate by mechanistic modelling because it depends on leaf unrolling, which shapes whorl geometry. In contrast, leaf emergence occurs when the leaf is hidden in the whorl and can be observed only by dissection, but can be simply calculated from blade and sheath lengths, which are usual variables of FSPMs. It is thus of interest to clarify whether leaf appearance or emergence is better correlated with the differentiation of the blade-sheath boundary.

In maize, the coordination between leaf appearance/emergence and sheath development holds only for juvenile leaves. The sheaths of upper leaves are initiated at a higher rate than lower leaves, which results in a decreasing duration of the blade extension with higher leaf position above the ear, and finally contributes to the progressive decrease in the blade size towards the top of the shoot (Andrieu *et al.* 2006; Zhu *et al.* 2014; Vidal *et al.* 2020). While a change in the rate of sheath development is well acknowledged, data describing sheath initiation in upper leaves are limited and of low accuracy. There is, thus, a gap in our understanding of the construction of the distribution of the blade size.

Another example of missing information is the rate of leaf initiation, which is an empirical parameter in most models, and is assumed to be constant throughout vegetative development. Studies on the apical meristem (Snow and Snow 1952; Douady and Couder 1996) relate the rate of leaf initiation to meristem diameter and primordium size, which both vary during vegetative development. Few experimental studies have been conducted that have precisely monitored leaf initiation in maize (reviewed in the discussion); therefore, assuming that the rate of leaf initiation is constant reflects a choice of simplicity rather than a well-established behaviour.

Models also need the relative extension rate (RER) during the exponential phase and the linear extension rate (LER) during the linear phase, which may be data-fitted (Zhu *et al.* 2014) or be regulated in relation to plant status in more deterministic approaches (Gauthier *et al.* 2020). Only a limited amount of data have been published that describe the pattern of RER or LER along a grass shoot. For maize, most of these data have been recently reviewed by Vidal *et al.* (2020).

While there is a consensus on the general patterns that describe the extension of grass leaves, only a few experimental studies have documented the initiation and extension of the full sequence of leaves along the shoot. Consequently, many unknowns are related to the ontogenetic changes that result in the sequence of leaf sizes along the shoot. In this study, we investigated the full sequence of initiation and extension of maize leaves in two cultivars with different leaf sizes originating from the same inbred line. We characterized the sequence of leaf and sheath initiation, coordination of the extension phases with emergences, and variation of the extension rate between phytomers. We then evaluated the assumptions on which the functional–structural models describing shoot development are based. In addition, we investigated the differences leading to the contrast in leaf sizes and the role of floral transition in the timing of sheath extension.

## Methods

**Figure 1.**
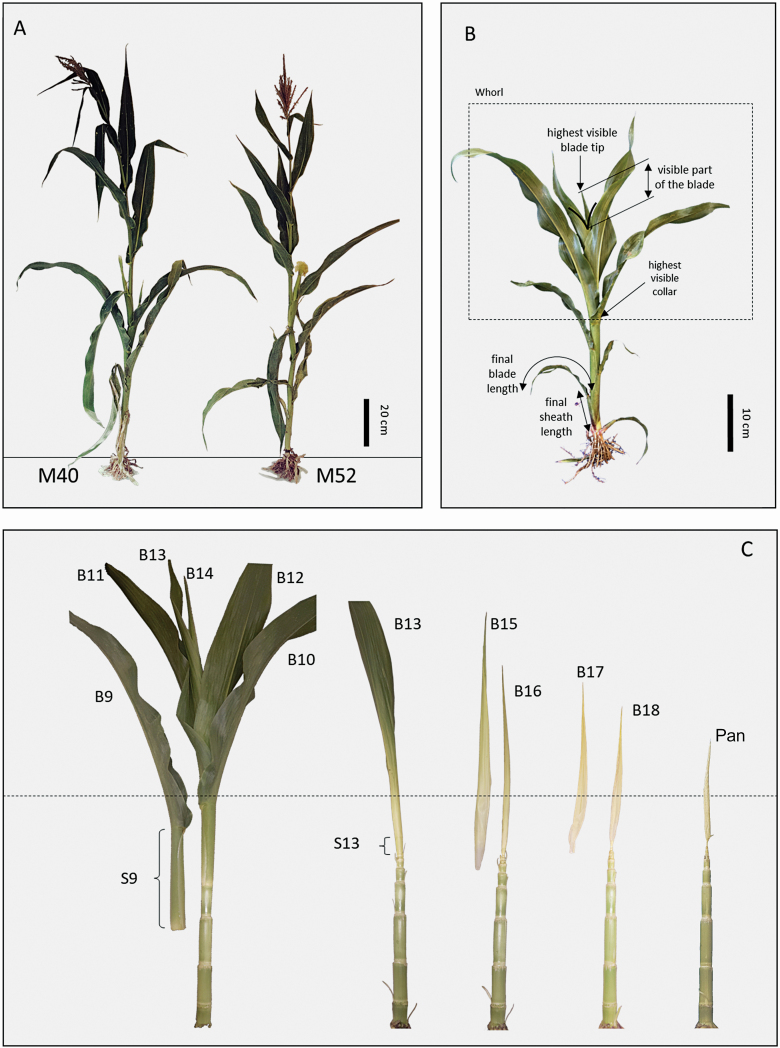
Architecture of maize cultivars M40 and M52. (A) Plants that have completed their vegetative development; M40 is taller and its female flowering is delayed of a few days compared with M52. (B) The non-destructive measurements. (C) A plant at successive stages of dissection. Bn and Sn refer, respectively, to the blade and the sheath of leaf n, Pan refers to the panicle. The horizontal dotted line shows approximately the height of the sheath tube. Leaves 1–10 are liguled, leaves 11–14 are visible, leaves 15–18 have emerged from the sheath tube but are not visible yet and are not exposed to direct light.

Two cultivars M52 and M40 were used in this study. Each of them independently derived by selfing and selection for the time of female flowering from the maize inbred line MBS847. They were chosen as representatives of the generation G13 of the Saclay’s divergent selection experiments for flowering time (Durand *et al*. 2010, 2015). M40 is later in female flowering compared with M52, but both cultivars are nearly synchronous for male flowering and typically form 18 or 19 leaves. M40 has a markedly larger leaf size than M52 (Fig. 1A).

### Agronomic design

The experiment was conducted in the field on the INRA campus at Thiverval-Grignon, near Paris, France. Seeds of cultivars M40 and M52 were sown on 26 April 2018, in plots (17 rows of length17.6 m) with a planting density of 9.54 plants m^–2^ and 80 cm between rows. A border of three rows was sown around each plot, with the same cultivars to avoid neighbouring effects on plant architecture. The surrounding field was sown with the maize commercial hybrid Konkordans (KWS SAAT SE & Co. KGaA, Einbeck, DEU). Weeds were controlled using 1.8 L/ha Dual Gold 960 EC (Syngenta, Bâle, CH) before seedling emergence and 0.25 L/ha Emblem Flo (Nufarm, Melbourne) plus 0.5 L/ha Callisto (Syngenta) on 31 may. Maize stem-borers were controlled using trichograms. Soil contained 40 kg N ha^–1^ at seedling emergence and crops received 160 kg N ha^–1^ before stem extension, ensuring no limitation of vegetative growth. Water stress was prevented by irrigating when necessary from June onwards.

### Plant growth monitoring

Destructive and non-destructive measurements from the time of seedling emergence to flowering were performed for both cultivars approximately three times per week. For all phytomers along the shoot, destructive measurements of the blade and sheath length from leaf initiation to maturity were used to build elongation curves. To ensure that elongation dynamics were representative of a median-sized plant, we sampled plants that had visible traits close to the median value of the population of plants. These median values were determined based on non-destructive monitoring of 30 reference plants. Data from non-destructive monitoring were also used to estimate the time of leaf and collar appearances.

For each phytomer position, organ extension models were fitted to experimental data from destructive samplings to estimate (i) the time of the blade and sheath initiations, (ii) parameters describing the kinetics of extension and final length, (iii) the time of emergence of leaf tips above the sheath tube and (iv) the time of male floral transition. In maize, tip emergence occurs while the leaf is fully hidden within the whorl and cannot be monitored by non-destructive measurements. Tip emergence was, therefore, estimated from the extension models fitted to the lengths of blades, sheaths and internodes, as the time when a leaf tip was above the highest collar. The floral transition was approximated as the time when the length of the apex reached 0.05 cm (Moncur 1981), and was determined by fitting an empirical model to measurements of apex length (destructive measurements) vs. thermal time.

The tip appearance, defined as the time when the leaf tip is exposed to an observer and to direct light, was monitored via non-destructive observations. Collars are visible as soon as they emerge from the sheath of the previous phytomer. Collar emergence was, therefore, assessed via both destructive and non-destructive observations. Final organ length was obtained via both destructive and non-destructive observations. Data from destructive and non-destructive measurements are provided in file SupData1-raw_data_s2.xlsx  **[****Supporting Information****]**.

#### Non-destructive monitoring.

At the centre of each experimental plot, we defined a rectangle of four rows by 8 m to be dedicated to non-destructive measurements until flowering. Thirty plants were randomly chosen at the seedling stage, and their leaves were regularly tagged to keep track of their rank along the stem. Two or three times per week, the rank of growing and mature leaves was noted and the exposed length of appeared blades was measured until their collar emerged (Fig. 1B). These data were used to estimate the decimal leaf stage as described below.

Once a collar had emerged, the final blade length was measured. Collar emergence was estimated from non-destructive observations (exposed blade length) as the mean of the thermal time at the last time where an emerged collar was not observed and the thermal time at the first time with an emerged collar.

In addition to providing data to monitor leaf stage and collar emergence, the measurements were used to define two criteria to be satisfied when sampling plants for dissection: (i) the length of the youngest mature blade $\left(L_{mature}^{youngest}\right)$ should be within the two central quartile of the distribution and (ii) the exposed length of the oldest growing blade $\left(L_{growing}^{oldest}\right)$ should be within 10 % of the median. At flowering, final dimensions of all non-senesced blades and sheaths were measured to verify that they were similar to plants from destructive measurements.

#### Destructive measurements.

At each sampling session, a new zone (1.6 × 1.6 m) was delimited within the part of the plot dedicated to destructive samplings. Visible dimensions of plants were measured in the field until sufficient plants (two to five depending on the plant growth stage) were found satisfying the criteria established from non-destructive samplings. In the laboratory, all visible dimensions were recorded on sampled plants. Plants were then dissected to measure the dimensions of young leaves and the apical meristem (Fig. 1C). Lengths greater than 1 cm were measured using a ruler, while those smaller than 1 cm were measured using a video microscope with ×50 to ×300 magnification (Hirox MX 5030RZ2, Tokyo, Japan)

#### Temperature and thermal time.

The extension dynamics of plant organs were expressed as a function of thermal time calculated by monitoring the temperature representative of the growing-zone. Before internode elongation, the growing-zone is located belowground; therefore, the temperature was measured using six thermocouples inserted in the soil at a depth of 3.5 cm. After internode elongation, the apex is located aboveground; thus, we used air temperature measured at 2 m height by meteorological station INRAE 78 615 003, distant 500 m from the experimental field. Thermal time was computed hourly using Equation (1):

$$\begin{array}{l} TT_{i}=\;\underset{j}{\sum }\;\max\left\{\frac{T_{j}-T_{b}}{24},0\right\} \end{array}$$ (1)

where $TT_{i}$ (°Cd) indicates the cumulated thermal time until hour *i* after sowing, $T_{j}$ is the average temperature for hour *j* after sowing and $T_{b}$ is the base temperature (here 10 °C). Thermal time is provided within the file SupData2-meteorological_data.xlsx  **[****Supporting Information****]**.

### Estimation of variables describing plant growth

#### Estimation of organ initiation.

Organ initiation $\left(t_{0,n}\right)$ was estimated from destructive measurements as the time when each organ reached a reference length $(L_{0,n}),$ corresponding to the smallest length at which organs could be accurately measured. This length was of 0.025 cm for blades and 0.1 cm for sheaths. To estimate the time when this length was reached, an exponential model was fitted to data collected for organs shorter than 1 cm, which were considered still in their meristematic phase. For the organ extension models described below, the initial length $L_{0,n}$ was taken as the reference length and the beginning of the first phase $\left(t_{0,n}\right)$ was set to the time estimated as described earlier.

#### Models of blade and sheath extension.

For each phytomer, we adjusted a model (Equations 2 and 2b) describing sheath and blade elongation in a succession of three phases, i.e. exponential, linear and plateau, as a function of thermal time. Models were fitted using the function ‘curve_fit’ in the ‘scipy.optimize’ package (Virtanen *et al.* 2020). These fits allowed estimating the times of the beginning of the linear phase and the end of growth but also rates of organ elongation and organ length at key stages of extension.

$$\begin{array}{l} L_{n,t}=\left\{ \begin{array}{ll} L_{0,n}.e^{r_{n}\left(t-t_{0,n}\right)} & if\;t_{0,n}\leq t\;\leq \;t_{1,n}\\ L_{1,n}+k_{n}(t-t_{1,n}) & if\;t_{1,n}\leq t\;\leq \;t_{2,n}\\ L_{2,n} & if\;t\;>t_{2,n} \end{array}\right. \end{array}$$ (2)

where $L_{n,t}$ is the length of the considered organ at time *t*; $t_{0,n}$, $t_{1,n}$ and $t_{2,n}$ are the times of initiation, the beginning of linear extension and the end of growth for phytomer *n*, respectively; and $r_{n}$ is the relative elongation rate of the considered organ, and $k_{n}$ is the linear elongation rate of the considered organ. $L_{0,n}$, $L_{1,n}$ and $L_{2,n}$ are the length of the considered organ at $t_{0,n}$, $t_{1,n}$ and $t_{2,n}$, respectively.

For blades, the visual observation of experimental data does not show a clear discontinuity of the rate of extension at the transition between exponential and linear phases; moreover, when fitting the model, we observed inconsistencies in the estimates of the time of the transition between exponential and linear growth. Consequently, we defined a constraint of continuity of the rate of extension at the exponential–linear transition (Equation 2b). We compared results when Equation 2 was fitted with or without considering the continuity constraint of Equation 2b.

$$\begin{array}{l} k_{n}=r_{n}.\;L_{0,n}.\;e^{r_{n}(t_{1,n}-t_{0,n})} \end{array}$$ (2b)

#### Tip and collar emergence and appearance.

Tip and collar emergence were computed using the dynamic of organ lengths calculated from the model fitted to experimental data. The emergence of an organ was considered the time when the tip of the organ (blade tip or collar) emerged from the highest collar in the plant. We characterized the appearance of a tip by the decimal leaf stage (DLS), which represents the decimal number of exposed leaves. The DLS was calculated following Andrieu *et al.* (2006) from the rank and exposed lengths of the two youngest exposed leaves of the plants monitored using non-destructive measurements.

$$\begin{array}{l} DLS=\;n+L_{e}(n)/L_{e}^{*}(n) \end{array}$$ (3)

where *n* is the number of exposed leaves, *L*_*e*_(*n*) is the exposed length of leaf *n*, and $L_{e}^{*}(n)$ is the exposed length of leaf *n* when leaf *n* + 1 appears.

## Results

### Patterns of mature architecture

**Figure 2.**
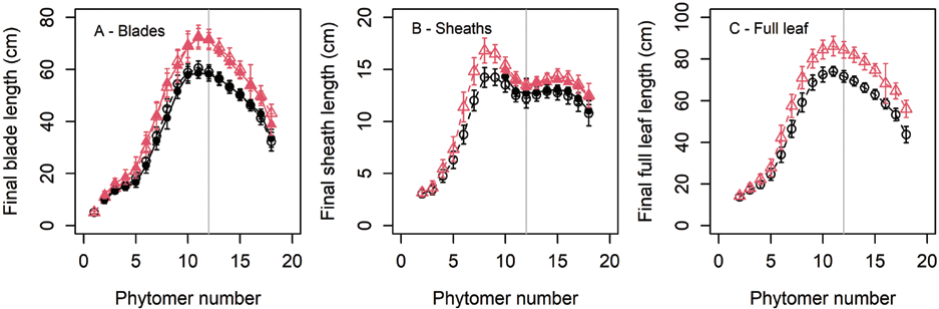
Distribution of the length of mature maize leaves. Red triangles correspond to cultivar M40, and black circles to cultivar M52. Empty symbols and dashed lines correspond to destructive data. Filled symbols with continuous lines correspond to non-destructive measurement data. Vertical grey lines indicate the position of the ear.

The majority (>85 %) of plants in both cultivars formed either 18 or 19 main stem leaves (mean = 18.3 in M52 and 18.7 in M40). The top ear was most frequently on phytomer 12 (mean = 11.9 in M52 and 12.3 in M40). Fig. 2 shows the distribution of the length of mature leaves along the stem. There was little difference (<1 cm) between plants of the same cultivar having 18 or 19 leaves, so for the sake of simplicity we pooled all plants independently of the final number of leaves. The distribution of leaf lengths showed the same patterns in both cultivars; however, the blades and sheaths were longer in M40 compared with M52. These patterns were also similar to those reported for cultivars Déa and Nobilis in Andrieu *et al.* (2006), with ear position being one rank above the longer leaf and coinciding with a local minimum in sheath length.

### Organ initiation

**Figure 3.**
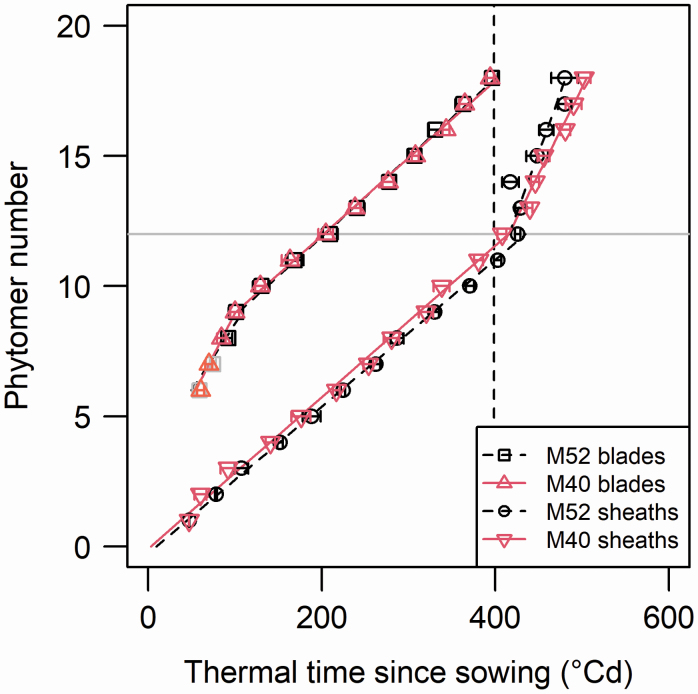
Time of initiation of blades and sheaths in maize cultivars M40 and M52. Symbols correspond to the time when each organ reached a reference length of 0.025 cm for blades and 0.1 cm for sheaths. Pale colours show blades of phytomer 6 and 7, for which calculating the time when they reached the reference length implied some extrapolation. The lines correspond to linear regressions fitted on the data. The horizontal grey line indicates the position of the ear. The dashed vertical line indicates the time of tassel initiation. Confidence intervals (95%) are shown as vertical bars and are smaller than the size of the symbols.

Fig. 3 shows the times when organs reached the reference length (0.025 cm for blades and 0.1 cm for sheaths). Leaves 6 and 7 were ~0.050 cm and ~0.025 cm long, respectively, at the first sampling date, so estimating the time of initiation of these leaves required slightly extrapolating the function fitted to length measurements vs. thermal time. The dynamics of the blade and sheath initiations showed the same patterns in both cultivars.

Blade initiation followed a relatively constant rate from blade 10 onwards, with a plastochron of 32.9 °Cd for M52 and 33.6 °Cd for M40. However, for leaves 6 through 9 the plastochron was markedly lower (19.7 °Cd and 15.3 °Cd, respectively). Even if some extrapolation was involved in the estimate of initiation of leaves 6 and 7, this was over a short period of their extension, and could not be the reason for the lower plastochron of leaves 6 through 9 (as shown from confidence intervals in Fig. 3).

Sheath initiation showed markedly different rates according to phytomer position. Specifically, phytomers below the ear had a plastochron of approximately 40 °Cd and those above the ear had a much faster plastochron of ~20 °Cd. As shown in Fig. 3, the change in the rate coincided with the initiation of the flag leaf.

### Organ extension

**Figure 4.**
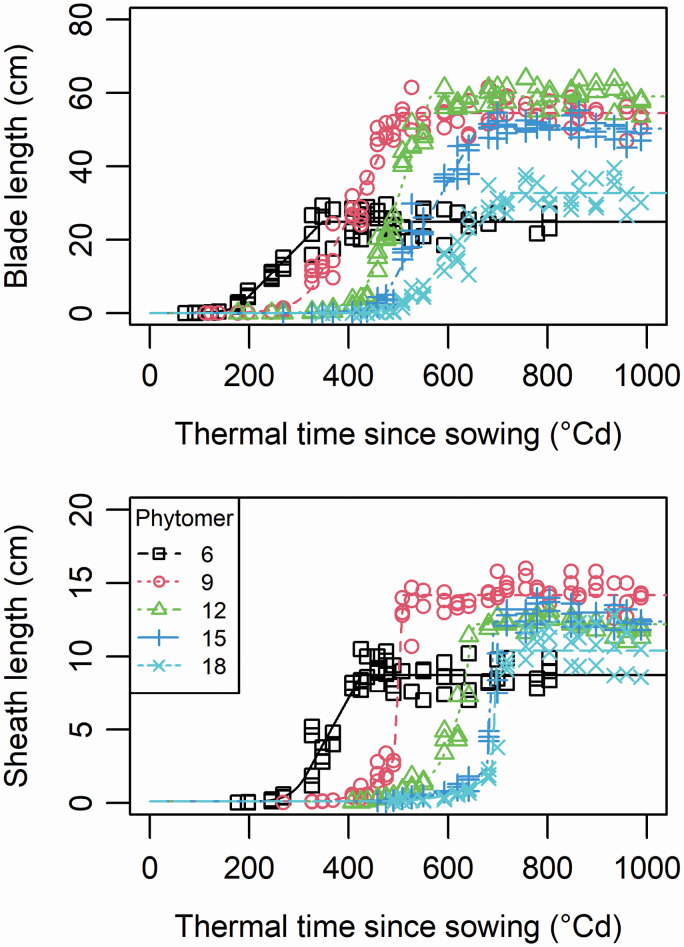
Measured and fitted dynamics of extension of (A) blades and (B) sheaths vs. thermal time for five phytomers along the stem of cultivar M52. Circles represent experimental data and lines show the adjustment with the three-phase model, from which RERs and LERs were estimated. Blade extension was fitted considering slope continuity between the experimental and linear phase.

Three phase models were fitted to experimental data of phytomers 4 to 18 as described in the Methods. This made it possible to estimate the extension rates during the exponential (relative elongation rate—RER) and linear phases (linear elongation rate—LER). The root mean square error (RMSE) ranged from 1.06 cm to 4.17 cm for sheaths and 1.79 cm to 5.38 cm for blades; as exemplified in Fig. 4, models adjusted to data without bias and RMSE primarily originated from plant-to-plant variability.

Fig. 4 shows the fit of the three-phase model (including exponential, linear phase and end of growth) for five phytomers along the stem in M52.The same data are shown in log scale in **Fig. S1**  **[****Supporting Information****]** to highlight the early stages of organ extension. Similar data and fits were obtained for phytomers 4 to 18 in both cultivars. The outputs of the model fitting are provided in file ‘SupData3-fit_3phase_models_s2.xlsx’ **[****Supporting Information****]**.

#### Blade extension

Identical estimates for RERs and near identical estimates for LERs were obtained whether or not the continuity constraint of the rate of leaf extension between the exponential and linear phase (Equation 2b), except for a few phytomers when the absence of constraint resulted in wrong estimates of the time of transition and LERs. Thus, considering the constraint of continuity did not introduce bias in the estimate of the extension rates; instead, it resulted in more robustness to noise in experimental data. Results will be presented for estimates obtained with this constraint of continuity of rate. **Fig. S2**  **[****Supporting Information****]** shows the comparison between both methods.

**Figure 5.**
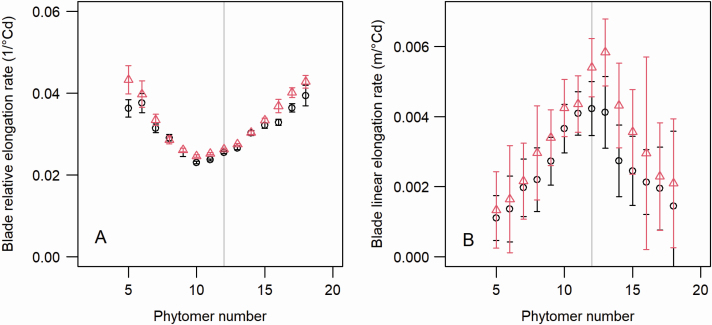
Blade relative (A) and linear (B) elongation rates for maize cultivars M40 and M52. Parameter values were estimated using a three-phase extension model with a slope continuity between the exponential and linear phases. Black circles correspond to M52, and red triangles indicate M40. The vertical grey line shows the position of the ear.

Leaf blade RERs were similar for both cultivars. They decreased from the lower phytomers to phytomers 10 to 11, and then increased for the upper phytomers (Fig. 5A). Leaf blade LERs showed the opposite trend, namely, increasing with higher phytomer position up to phytomer 11 (M52) or 13 (M40) and decreasing for phytomers above the ear (Fig. 5B).

The pattern of RER shown here markedly differs from those reported in Vidal *et al.* (2020), which showed a plateau or a slow decrease for phytomers above the ear. The experimental protocol and data analysis were the same in this study and in Vidal *et al.* (2020); therefore, the differences observed represent variations of plant behaviour relating to genotype and/or environmental conditions.

Although the distribution of RER showed the same pattern in both cultivars, there was a trend for higher values in M40 compared with M52. LERs were especially higher in M40 compared with M52. In contrast, the duration of linear extension was similar, or lower, in M40 compared with M52 (depending on phytomer, see **Fig. S3**  **[****Supporting Information****]**): despite a mostly lower duration of extension, the higher LERs of blades in M40 determined their greater length compared with M52.

#### Sheath extension.

**Figure 6.**
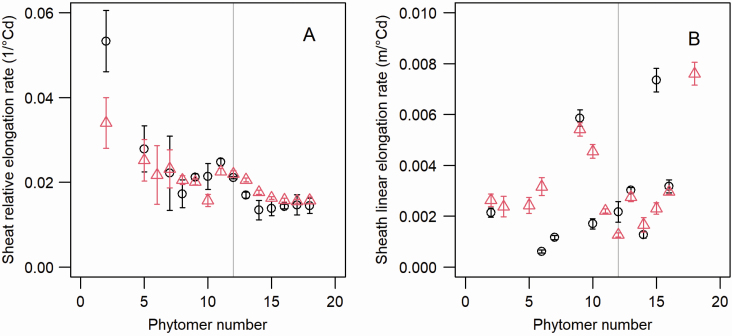
Sheath relative (A) and linear (B) elongation rates for maize cultivars M40 and M52. Black circles correspond to M52, and red triangles correspond to M40.

The RER of the sheath decreased (Fig. 6A) for successive phytomers, then plateaued for phytomer 14 and above. The data showed similar values for both cultivars. Sheath LERs showed variability, reflecting the low accuracy of the estimates. As illustrated in Fig. 4B, the estimated durations of linear sheath extensions were variable (from 8.8 to 95.0 °Cd). Typical values for the time interval between dissections ranged from 20 to 30 °Cd, which implies that sampling dates were too spaced to allow for an accurate characterization of the linear phase of sheath extension.

### Emergence events and their relation with organ elongation

**Figure 7.**
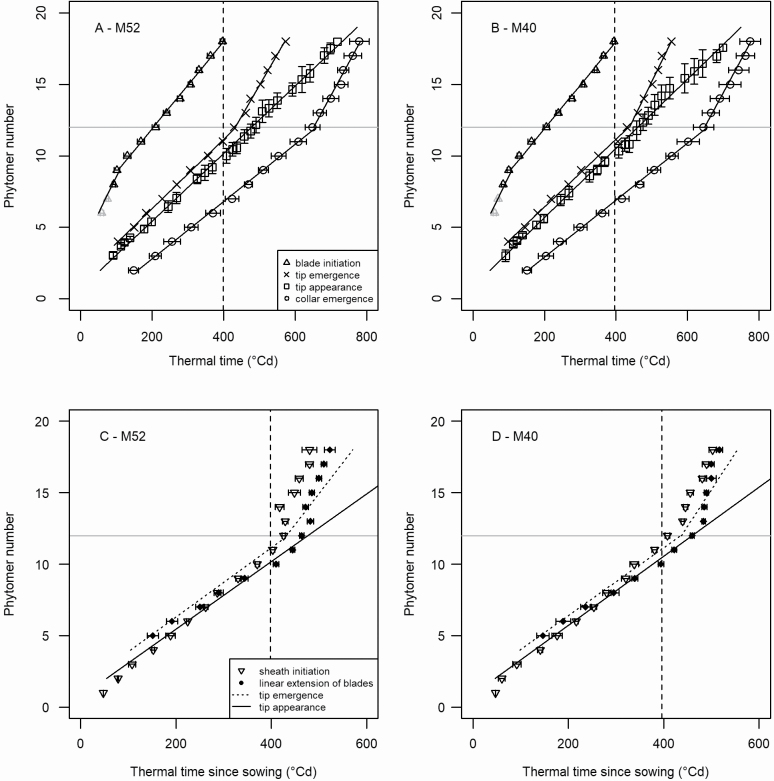
Emergence events: tip emergence and appearance, collar emergence, beginning of blade linear extension and sheath initiation. The dotted vertical line shows the time of tassel initiation. (A and B) Upwards-facing triangle: leaf initiation (blade length = 0.025 cm); cross: tip emergence; square: tip appearance; circles: collar emergence. Lines correspond to linear models adjusted on points in (A) and (B). (C and D) Downwards-facing triangles: sheath initiation (sheath length = 0.1 cm); filled diamonds: linear extension of blades. Lines correspond to the tip emergence and appearance as fitted on data shown in (A) and (B).

The rate of tip appearance was relatively constant (Fig. 7A and B), except for a transitory increase around the appearance of phytomers 10–15. When calculated using a linear model (R^2^ > 99.5 %), the phyllochron was estimated to be 42.3 °Cd for M52 and 41.4 °Cd for M40 (Table 1). For lower phytomers, tip emergence occurred shortly before the tip appearance. The interval between these two events increased slowly until phytomer 12, and then more abruptly, reflecting the increase in the length of the whorl formed by growing leaves. Sheath initiation and collar emergence occurred at a lower rate for phytomers 1 through 12 and a higher rate for phytomers above the ear. The change was even more marked than for tip emergence.

**Table 1.** Thermal time intervals (°Cd) between the initiation, emergence, and appearance events on successive phytomers. Values into brackets define confidence intervals.

	M52		M40	
	Lower phytomers	Upper phytomers	Lower phytomers	Upper phytomers
Blade initiation	19.7 (±3.2)	32.9 (±0.6)	15.3 (±1.3)	33.6 (±0.7)
Sheath initiation	35.5 (±0.4)	10.9 (±2.1)	34.3 (±0.75)	15.0 (±1.2)
Tip emergence	41.2 (±0.5)	23.2 (±0.8)	42.2 (±0.8)	19.6 (±1.1)
Tip appearance	42.3 (±0.4)		41.4 (±0.6)	
Collar emergence	50.0 (±0.8)	21.8 (± 1.2)	50.2 (±0.8)	21.6 (±1.0)

The beginning of linear extension of the blade occurred simultaneously with tip emergence for the lower phytomers, (Fig. 7C and D) but tended to be increasingly delayed for higher phytomers up to the ear position, where it occurred approximately simultaneously with the tip appearance. The initiation of sheaths showed the opposite behaviour, occurring approximately synchronously with the tip appearance for lower phytomers and with the tip emergence for phytomers near the ear. Ear position marked a sharp increase in the rate of sheath initiation and of the start of linear blade extension. For upper phytomers, the rate of sheath initiation was higher than the rate of tip emergence, and thus of blade initiation. This resulted in that both the exponential and linear phases of blade extension were increasingly shortened for phytomers above the ear.

### Correlations between the size of successive phytomers

**Figure 8.**
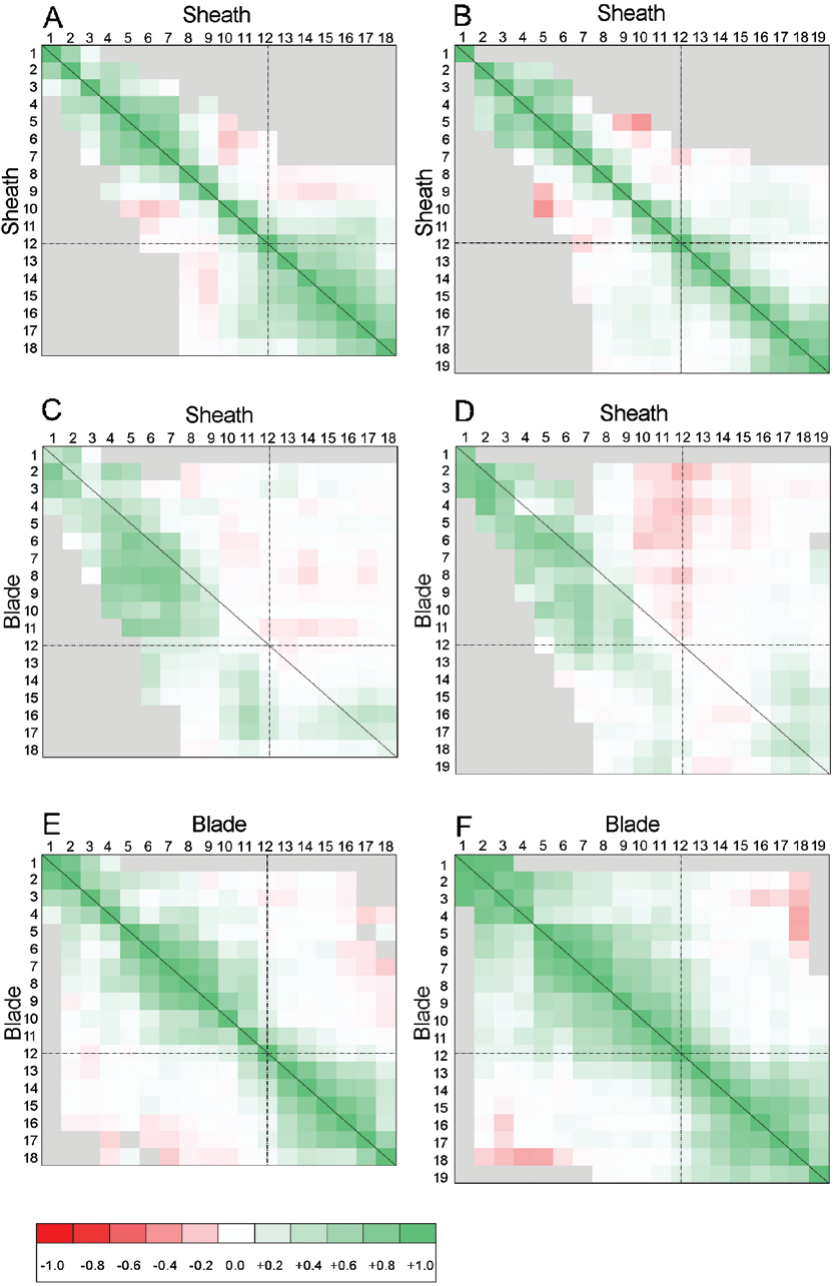
Correlations between the final length of blades and sheaths along the maize shoot. The source of variation is plant-to-plant variability within two sets of 30 plants from cultivars M52 (left) and M40 (right). For phytomer positions given by the coordinate of the pixel, the colour of a pixel represents the *r*^*2*^ between the length of blades and/or sheaths of a same shoot. Colour chart is at the bottom of the figure, positive values of *r* are associated with green colour and negative values of *r* are associated with red colours, grey pixels mean that less than 10 entities were available and the correlation was not calculated. The horizontal and vertical discontinuous lines show the position of the ear.

We used the two sets of 30 plants from the non-destructive measurements to analyse the correlation existing between the length of mature blades and sheaths along the shoot (Fig. 8). Similar patterns were found in both cultivars. The lengths of two successive sheaths (Fig. 8A and B) or two successive blades (Fig. 8E and F) were strongly correlated (*r*^*2*^ ~0.7 to 0.9); these correlations held over a variable number of phytomers depending on their position along the shoot. At the bottom of the plant (phytomers 3 to 7), and to a lesser extent at the top of the plant (above ear position), correlations held up three to five phytomers. Conversely, no correlations existed between the size of vegetative phytomers and that of phytomers above the ear, meaning that small (or large) plants in early stage did not preferentially result in small (or large) plants at flowering. The correlation between sheaths and blades (Fig. 8C and D) also had noticeable patterns. Specifically, for bottom phytomers, strong correlations existed between the length of the sheath and the length of the blades from the same or upper leaves; these correlations frequently held up six successive phytomers (*r*^*2*^ between lengths of sheath *n* and blade *n* + 6 being >0.4). Interestingly, the length of a sheath was more highly correlated with the length of the blade of phytomer *n* + 1 than with that of the same phytomer. The strong correlation that exists between the length of the sheath tube and the final length of the blades is well recognized for grasses at a vegetative stage and is explained by the dependency of the size of the leaf growth zone on the size of the sheath tube that protects it from the environment (Kemp 1980). Phytomers above ear position showed weak or no correlations between sheath and blade dimensions (Fig. 8C and D). Similarly, the length of upper leaf blades was also largely independent of the length of the sheaths of the phytomers below the ear. This lack of correlation suggests that, for phytomers above ear position, the length of the sheath tube played little role in the final length of the associated blade. This is consistent with results in Vidal *et al.* (2020), which showed a lack of correlation between the length of the elongation zone in upper leaves and the length of the sheath tube.

## Discussion

The two cultivars investigated differed by the larger leaf size in M40, which was as expected, but had the same pattern of variation of final length of leaves with phytomer position (Fig. 2). The dynamics of sheath and blade initiation, extension, and emergence, were identical between the two cultivars, but differed by slightly higher RERs and significantly higher LERs in M40, which produced marked differences in leaf lengths. A high degree of similarity is consistent with the fact that both cultivars share the same genetic background and were grown under the same conditions. Moreover, identifying highly similar dynamics from two independent datasets gives confidence in the robustness of the results.

### Blade initiation and plastochron

The change in the plastochron during ontogeny was unexpected. In both cultivars, the time interval between primordia reaching the reference length (0.025 cm) was ~20 °Cd for leaves 6 to 9 and ~33 °Cd for leaves 9 to 18. The plastochron is usually assumed to be constant during the initiation of maize vegetative primordia. However, few studies have characterized the full sequence of successive leaves.

Some previous studies have used air temperature as a reference, which may not accurately reflect the temperature of the apical meristem. For example, Abbe (1951), Warrington and Kanemasu (1983) and Zur *et al.* (1989) reported an increasing, stable and a decreasing rate of initiation for successive phytomers, respectively. Leaves are initiated while the apical meristem is below the soil surface; therefore, soil temperature is recognized as an accurate proxy for the temperature driving development at this stage (Cellier *et al.* 1993; Bonhomme 2000), which is why soil temperature was measured in this study. However, data on the initiation rate using soil temperature are rare and we found no studies that documented the first post-embryonic leaves in maize using soil temperature.

Padilla and Otegui (2005) reported a linear relationship between the numbers of initiated leaves (above eight) and appeared leaves for a set of 16 maize cultivars; they found a total of approximately 1.6 initiated primordia per appeared leaf. A linear relationship also existed in our experiment, but the rate we observed was lower (1.4 primordia/appeared leaf). In sorghum, Lafarge and Tardieu (2001) reported a constant plastochron across plant development, while Clerget *et al.* (2008) observed a marked change, with leaves above 20 being initiated at a rate half that of lower leaves. Thus, the limited number of existing studies shows that both linear and bilinear behaviours do exist, but we found no information specific to the first post-embryonic leaves, which were found here to be initiated with a higher rate.

In our work, we characterized plastochron as the time interval between successive primordia reaching 0.025 cm, which may differ from the time interval between the true initiation of leaves because leaves are initiated at a smaller size (~0.005 cm). The time taken to reach 0.025 cm is in the range of 40 °Cd and may vary between phytomers, depending on the early growth rate and variations in the initial size. Our decision to characterize plastochron as described earlier was motivated by two reasons. First, our estimate could be calculated with high accuracy, whereas characterizing plastochron based on simple counts of primordia is hampered by the uncertainty in the time elapsed between actual initiation and counting. Second, our estimates are directly meaningful in the perspective of defining models of leaf extension, for which initial conditions must be specified both in terms of time and dimension. Such models usually consider a constant plastochron and the same initial length for all phytomers, which was not the behaviour observed in our experiment and may be the reason for the underestimation of the size of the basal leaves reported in Vidal *et al.* (2020). Finally, little data are available describing the full sequence of initiation of successive phytomers; our results provide original data for early phytomers, which complement existing evidence for the upper phytomers (Clerget *et al.* (2008) that the plastochron may change during vegetative development. A deeper knowledge of plastochron changes is useful to develop functional–structural plant models that simulate the whole sequence of phytomer growth along a shoot.

### Sheath initiation, leaf emergence and leaf appearance

This study provides novel information on the sequence of sheath initiation. Similar to what was done for leaf primordia, we characterized sheath initiation by the time when sheaths had a reference length of 0.1 mm. In both genotypes, sheaths of phytomers 1 to 12, i.e. below the ear position, were initiated at a constant rate (~35 °Cd). The boundary between the sheath and blade was first observed at leaf appearance for the lower phytomers, but coincided with leaf emergence for phytomers 9 to 10 (Fig. 7C and D). Thus, the coordination we identified in this study is similar to that reported in previous studies (Skinner et Nelson 1994, 1995; Arredondo and Schneider 2003; Fournier *et al.* 2005; Birch *et al.* 2007; Zhu *et al.* 2014; Vidal *et al.* 2020) but neither emergence nor appearance was a perfect candidate.

Fig. 7C and D illustrates two empirical features of high practical value that should be confirmed in further experiments. First, leaf appearance followed leaf emergence with a nearly constant delay of ~30 °Cd. Leaf appearance is readily monitored through non-destructive experiments but is difficult to simulate in mechanistic models because this requires an accurate description of the whorl geometry across time. Conversely, emergence can be readily simulated from models of leaf extension, but is difficult to characterize experimentally because it occurs within the whorl. Thus, our observation of a constant delay provides a way to bridge an important gap between model-related and measurable variables. Second, the timing of sheath initiation of lower leaves could be consistently described as following a linear progression with thermal time, synchronized with tip emergence for the lower phytomers (leaf 4 or 5) and with tip appearance for the ear leaf. This transposes to species with a large whorl, a finding established mainly for species with a short whorl.

The rate of sheath initiation changed markedly at tassel initiation (400 °Cd), which affected phytomers above the ear. Such an abrupt change in the rate of sheath initiation was previously reported by Andrieu *et al.* (2006) and Birch *et al.* (2007) and is an important feature in models predicting the pattern of blade lengths along the shoot (Zhu *et al.* 2014; Vidal *et al.* 2020). However, in the cited models, it was assumed that sheath initiation for upper leaves would occur at the same rate as leaf initiation, which was assumed to be the same for all phytomers. Here, we observed that the mean time interval between successive sheaths reaching the reference length of 0.1 cm was ~20 °Cd, much shorter than the plastochron of the corresponding leaves (~33 °Cd). This observation suggests that underestimating the rate of sheath initiation in upper phytomers was the reason for the previous overestimation of blade length in upper leaves (Zhu *et al.* 2014).

Finally, our results confirm a close coordination between sheath initiation and tip emergence or appearance in juvenile leaves, which plausibly reflects a response to environmental triggers perceived by leaf tips. Our findings also confirmed a much higher rate of sheath initiation in upper phytomers. However, the data collected in this study show a more complex reality than what has been represented in previous models. Specifically, sheath initiation followed uneven kinetics, where lower sheaths were initiated at an intermediate time between leaf emergence and appearance, and higher sheaths were initiated at a higher rate than leaves. Very little experimental data are available in published literature, so we conclude that identifying robust rules for sheath initiation in upper phytomers remains an important step for mechanistic prediction of the distribution of leaf length along a shoot.

### Dynamics of leaf extension

We also obtained novel results on the dynamics of leaf extension. An interesting methodological aspect of this study was the high consistency of estimates of RERs and LERs when the leaf extension model was fitted with or without assuming a continuity constraint. Both methods yielded identical results, except for a few phytomers for which noise in experimental measurements impacted the estimate of the exponential–linear transition (and thus the LER estimates) when exponential and linear rates were estimated independently. Considering a continuity constraint to describe the kinetics of the blade extension with three parameters instead of four, increased the robustness of parameter estimates. This is evident from the smooth pattern of LER vs. phytomer position (Fig. 5B) and its consistency between the two genotypes.

When estimated with the continuity constraint, the distribution of LERs vs. phytomer position showed a similar pattern in both genotypes, namely, increasing up to ear position then decreasing, which was as expected from previous studies (Andrieu *et al.* 2006, Birch *et al.* 2007  Song *et al.* 2016). M40, however, differed from M52 with higher values of LERs. In contrast, the duration of linear extension was similar, so LERs primarily explained the difference in blade length between the two cultivars. The distribution of RERs was highly similar in both genotypes. However, this pattern, with a minimum for median phytomers, differed markedly from previously published data, which showed a plateau or slow decrease of RER for upper phytomers (Vidal *et al.* 2020). Future studies should be conducted to determine whether the variation is linked to genotypic differences (the cultivars used here originated from inbred lines, while former studies used hybrids), growth conditions (plant density in this experiment was lower than in previous studies) or both.

### Correlations between final organ size dimensions

As expected in a field experiment, there was a variability in size between plants; plants were similar in the relative size of components, indicating strong correlations between the length of the sheath and that of the blade in a mature phytomer and between sheath or blade lengths in successive phytomers. Among other mechanisms involved in building these correlations, there is the impact of the size of the sheath tube on that of the growth zone (Kemp 1980) and ultimately on the length of the blade and sheath of the enclosed phytomers (Casey *et al.* 1999). Vidal *et al.* (2020) proposed that the profound change in sheath initiation after floral change should impact these correlations; therefore, we tested this hypothesis by a systematic analysis of the system of correlations along the shoot. The more striking result was obtained when analysing the relationship between sheaths and blades. For vegetative phytomers, we found strong correlations between the length of sheaths and blades of up to five or six sequential phytomers. However, this pattern was not found for upper phytomers. Mature lengths of blades above the ear were not correlated with sheath lengths, even within the same phytomer. When considering the length of successive blades or successive sheaths, variations in the correlation along the shoot were less distinct than between the sheath and blade. Correlations held up several phytomers, either vegetative or reproductive; it was noticeable, however, that the correlation was weakly propagated around ear position, thus the link between the size of phytomers below the ear and that of phytomers above the ear was weak. Variability in plant size existed in both the early and late stages of development, but the small plants (or large plants) in the early stages were not necessarily the small plants (or large plants) at flowering.

## Conclusion

This study showed that the final leaf length in two cultivars with a common genetic background originated from differences in leaf extension rates; the patterns identified for extension dynamics and coordination were identical in the two cultivars. This supports the robustness of such patterns, allowing contrasting phenotypes. Thanks to detailed measurements on the early phases of plant growth combined with non-destructive measurements at the population scale, this work provided novel information on the dynamics of leaf and sheath initiation that will be useful to improve plant models.

## Supporting Information

The following additional information is available in the online version of this article—

**SupFigures.docx.** Supplementary Figs. S1–S3 with legends.

**SupData1-raw_data_s2.xlsx.** contains all raw data collected in the experiment.

**SupData2-meteorological_data_s2.xlsx.** Meteorological data.

**SupData3-fit_3phase_models_s2.xlsx.** Value and confidence intervals of the parameters of models of blade and sheath extension.

**SupData4-key_events_organ_elongation_s2.xlsx.** Values for DLS and collar emergence vs. thermal time.

plaa072_suppl_Supplementary_Figures

plaa072_suppl_Supplementary_Data_1

plaa072_suppl_Supplementary_Data_2

plaa072_suppl_Supplementary_Data_3

plaa072_suppl_Supplementary_Data_4

## Notes

**Form & Function. Chief Editor:** Kate McCulloh
