# Supplementary material for: Two maize cultivars of contrasting leaf size show different leaf elongation rates with identical patterns of extension dynamics and coordination
Source: AoB Plants. 2021 Jan 4;13(1):plaa072. doi: 10.1093/aobpla/plaa072 (PMC7877697; doi:10.1093/aobpla/plaa072)
Supplement: plaa072_suppl_Supplementary_Figures [file plaa072_suppl_supplementary_figures.docx]

**Supplementary figures to paper:** Two maize cultivars of contrasting leaf size show different leaf elongation rates with identical patterns of extension dynamics and coordination.

Published in AoBPlants

**Authors:** Tiphaine Vidal, Hafssa Aissaoui, Sabrina Rehali, Bruno Andrieu*

UMR ECOSYS, INRA, AgroParisTech, Université Paris-Saclay, 78850 Thiverval-Grignon, France

* For correspondence. E-mail [bruno.andrieu@inrae.fr](mailto:bruno.andrieu@inrae.fr)


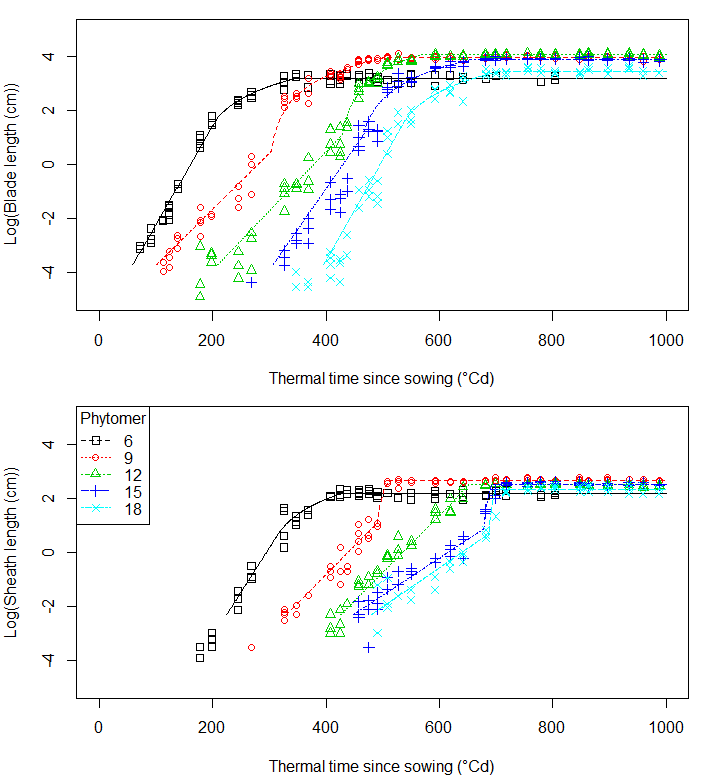


Figure S1: Measured and fitted dynamics of blade (a) and sheath (b) lengths *vs* thermal time for five phytomers along the stem of maize cultivar M52. Data are the same as in Fig3, but lengths are shown here with logarithmic scale. Symbols show experimental data and lines show the adjustments with the three-phase model, from which RERs and LERs were estimated with slope continuity.


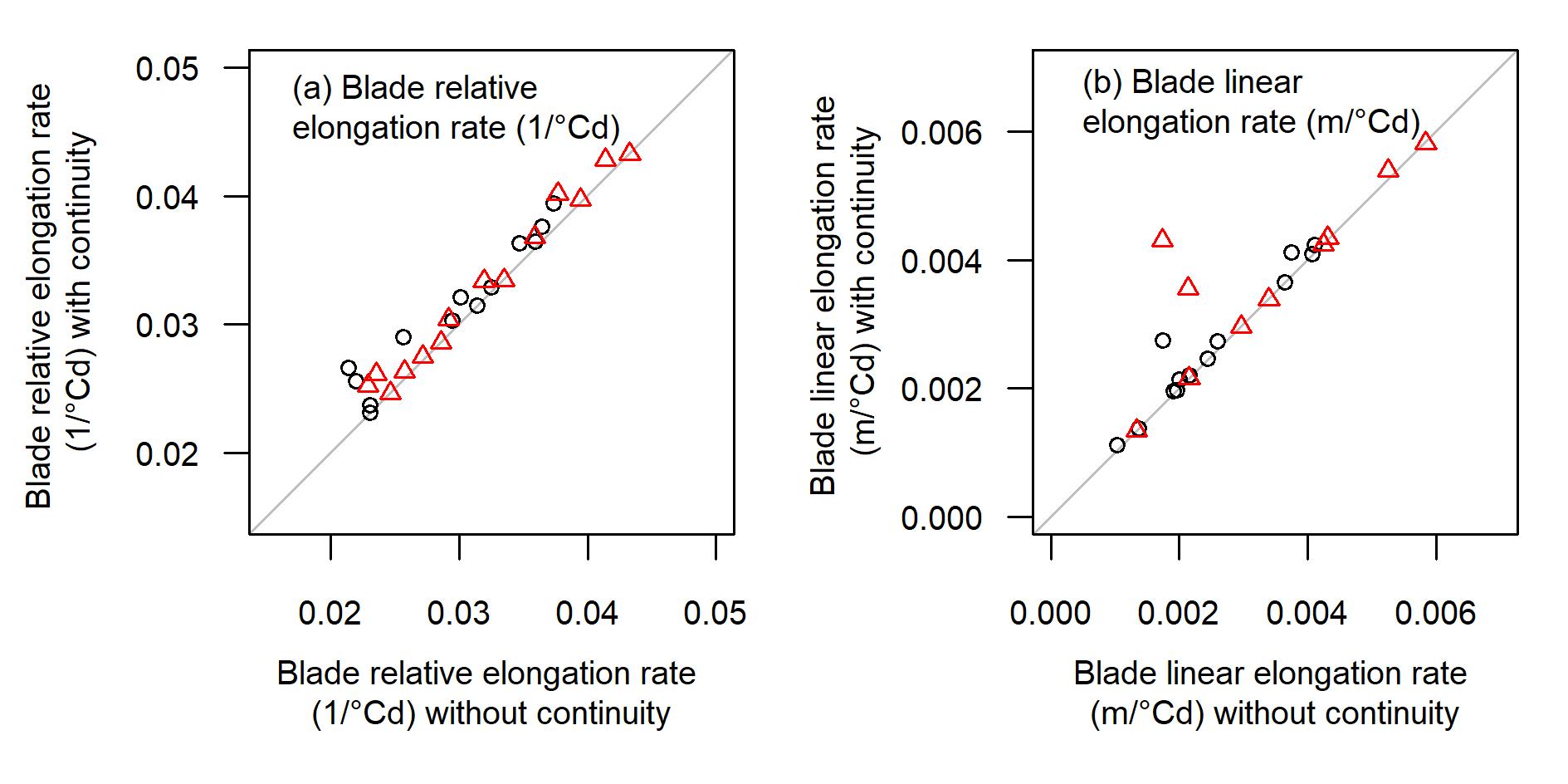


Figure S2: Blade relative and linear elongation rates computed with or without assuming slope continuity in blade extension, for maize cultivars M40 (red triangles) and M52 (black circle).


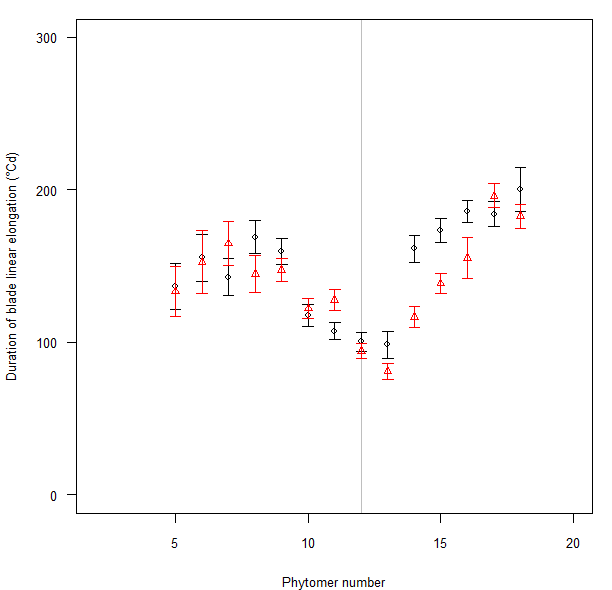


Figure S3: Duration of blade linear phase of extension, for maize cultivars M40 (red triangles) and M52 (black circles).
